# Redox properties of quercetin iron II complex with enhanced antioxidant and antiviral activities

**DOI:** 10.1038/s41598-025-31433-z

**Published:** 2025-12-19

**Authors:** Sara E. Abdel Hameed, Weam M. Abou El-Maaty, Esam A. Gomaa, Fathi S. Awad

**Affiliations:** Chemistry Department Faculty of Science, Mansoura University, Mansoura, Egypt

**Keywords:** Iron(II)-quercetin complex, Cyclic voltammetry, SARS-CoV-2 spike protein, Biochemistry, Cancer, Chemical biology, Chemistry, Computational biology and bioinformatics, Drug discovery

## Abstract

Quercetin is a naturally occurring flavonoid widely recognized for its potent antioxidant, anti-inflammatory, and anticancer effects. However, its clinical application is limited by low water solubility and poor bioavailability. In this study, the complexation of quercetin with iron (II) ions was systematically investigated through electrochemical, spectroscopic, biological, and computational techniques to improve its physicochemical and pharmacological performance. Cyclic voltammetry revealed that quercetin undergoes irreversible oxidation due to semiquinone and para-quinonemethide formation, while Fe(II) exhibited quasi-reversible redox behavior, which became less reversible upon complexation, suggesting altered electron transfer kinetics. The stability constant (log βMX = 3.29) and the negative Gibbs free energy (ΔG = − 19.07 kJ/mol) confirmed spontaneous and thermodynamically favorable complex formation. UV–Vis spectroscopy showed significant bathochromic shifts (Δλ = 66 nm), and the continuous variation method established a 1:1 metal-to-ligand stoichiometry. ¹H NMR spectroscopy and X-ray diffraction patterns further confirmed the coordination sites and structural modifications upon metal binding. The Fe(II)-quercetin complex demonstrated superior antioxidant capacity in DPPH radical scavenging assays, with an IC₅₀ of 21.86 µg/mL compared to 23.47 µg/mL for free quercetin, confirming enhanced radical scavenging activity upon complexation. Cytotoxicity assays on HepG2 and MCF-7 cancer cell lines revealed enhanced bioactivity of the complex, with IC₅₀ values of 26.80 and 18.60 µM, respectively. Antifungal testing showed moderate inhibition against Candida albicans. Molecular docking simulations using the SARS-CoV-2 spike protein (PDB: 7JWY) indicated stronger binding affinity for the complex (− 5.42 kcal/mol) than quercetin alone, suggesting potential antiviral efficacy. Additionally, DFT calculations revealed increased softness and electrophilicity of the complex, supporting its enhanced reactivity. Collectively, these findings position the Fe(II)-quercetin complex as a promising multifunctional agent with potential applications in antioxidant therapy, cancer treatment, and antiviral drug development.

**Supplementary Information:**

The online version contains supplementary material available at 10.1038/s41598-025-31433-z.

## Introduction

 The electrochemical method known as cyclic voltammetry (CV) is highly effective widely employed to investigate redox behavior, elucidate complexation mechanisms, and characterize the electrochemical properties of molecular species that have been adsorbed onto electrode surfaces or in solution^1,2^. Utilizing a three electrode setup—which includes a counter electrode, reference electrode, and working electrode —CV enables the analysis of electron transfer kinetics and thermodynamic parameters by examining peak potentials and currents^3,4^. Through the modulation of experimental conditions, such as scan rate and analyte concentration, valuable insights into complex formation, stability, and reaction reversibility can be obtained^5^. –^6^Furthermore, CV serves as an essential analytical tool in diverse scientific fields, including environmental, materials, and bioinorganic chemistry, owing to its utility in probing metal-ligand interactions and redox-active centers^7–9^.

Quercetin is a naturally occurring polyphenolicflavonol found in large quantities in various fruits and vegetables, including apples, onions, berries, and kale^10,11^. It possesses a comprehensive spectrum of biological activities, as well as antioxidant, anti-inflammatory, anticancer, and cardio-protective effects. These benefits are largely attributed to its free radical scavenging capability and interaction with key biological pathways. However, quercetin suffers from limited aqueous solubility and poor bioavailability, which hinder its therapeutic potential. To overcome these limitations, metal complexation has occurred as a promising strategy to enhance the physicochemical and pharmacological properties of quercetin^12–23^.

Iron(II), a transition metal ion known for its biological relevance and redox activity, has demonstrated notable antimicrobial and potential antiviral effects, including activity against enveloped viruses such as SARS-CoV-2. The formation of iron(II)-quercetin complexes may not only improve quercetin’s solubility and stability but also introduce synergistic effects that enhance its antioxidant, cytotoxic, and antiviral performance. In particular, studies have shown that iron can disrupt viral envelopes and impair viral replication, supporting its utility in antiviral therapy^24^^25^..

SARS-CoV-2, the connective agent of the COVID-19 pandemic, employs its spike protein especially the (RBD) receptor-binding domain to engage the receptor for the human angiotensin-converting enzyme 2 (ACE2), which makes viral entrance easier. The spike protein structure denoted by PDB ID: 7JWY is of particular interest due to its pH-dependent conformational states and binding behavior^26,27^. Molecular docking studies targeting this protein can provide predictive insights into the potential inhibitory interactions of small molecules, aiding the discovery of new therapeutic candidates^28^.

In this study, we explore the electrochemical performance of quercetin and its iron(II) complex using cyclic voltammetry and UV–Vis spectroscopy, assess their antioxidant and cytotoxic activities, and explore their potential antiviral properties via molecular docking against SARS-CoV-2 spike protein. By integrating physicochemical characterization with biological evaluation, this study aims to highlight the multifunctional prospective of the iron(II)-quercetin complex and contribute to the enlargement of unique metal–flavonoid therapeutics with enhanced bio-efficacy.

## Experimental and computational section

This section includes both experimental methodologies applied to characterize the Fe(II)-quercetin complex and computational approaches, including molecular docking simulations, to investigate theoretical interactions and binding affinities.

### Chemicals

2−(3,4 − dihydroxyphenyl) − 3,5,7 − trihydroxy − 4 H − 1−benzopyran − 4−one is a chemical formula of quercetin showed in (Fig. S1), ferrous ammonium sulfate (NH_4_)_2_SO_4_⋅FeSO_4_⋅6H_2_​O, potassium chloride (KCl), and high-purity ethanol (≥ 99.9%) were all procured from Sigma-Aldrich and used without auxiliary purification. Stock solutions of (NH_4_)_2_SO_4_.FeSO_4_.6H_2_O and KCl were prepared at a concentration of 0.1 M using freshly distilled deionized water with a conductivity of approximately 6 µS/cm. Quercetin was dissolved in ethanol to prepare a 0.01 M solution. All solutions were prepared using high-purity distilled water and stored in amber glass containers to avoid light-induced degradation.

### Solutions

All solutions were prepared at room temperature. In ethanol, quercetin was dissolved to obtain a 0.1 M solution and stirred continuously for 10 min to ensure completely soluble. Aqueous solutions of ferrous ammonium sulfate (NH4)_2_SO_4_⋅FeSO_4_⋅6H_2_O and potassium chloride (KCl), with 0.1 Mconcentration, were equipped using distilled water and similarly stirred for 10 min. All solutions were deposited in amber glass vials to protect them from light and maintain chemical stability. Prior to use, each solution was examined for clarity, homogeneity, and concentration accuracy.

### Cyclic voltammetry measurements

Cyclic voltammetry (CV) measurements were carried out using a DY 2100 cyclic voltammeter over a potential range from + 1.5 to − 1.5 V at various scan rates and analyte concentrations. A conventional three-electrode system was employed, consisting of a glassy carbon working electrode, a platinum wire auxiliary electrode, and an Ag/AgCl reference electrode immersed in saturated KCl. The working electrode was carefully polished with fine alumina slurry (Al₂O₃), rinsed with deionized water, and dried to ensure a clean and reproducible surface. A 0.1 M potassium chloride (KCl) solution was used as the supporting electrolyte. Ethanol was selected as the solvent due to the high solubility of quercetin, as it does not interfere with the electrochemical behavior of the Fe(II)-quercetin complex. Prior to each measurement, the test solutions were purged with high-purity nitrogen gas for 10 min to remove dissolved oxygen. Nitrogen flow was maintained during the experiments to ensure an oxygen-free environment and prevent interference with redox processes.“The concentration of the Fe(II)-quercetin complex used for diffusion coefficient calculation was determined from its UV–Vis calibration curve and adjusted to the final known concentration used in the electrochemical cell, which based on the initial stoichiometric ratio of Fe²⁺ to quercetin (1:1). The complex solution was prepared by mixing equimolar amounts of ferrous ammonium sulfate and quercetin, and the concentration was calculated from the limiting reagent, assuming complete complexation. This concentration was then used in the Randles–Sevcik equation to evaluate the diffusion coefficient.”

### Pharmacology

#### Cytotoxic activity of quercetin, Iron(II), and Iron(II)-quercetin complex

##### Cell lines and culture conditions

Two human tumor cell lines were used to assess the investigated drugs’ cytotoxic activities: breast adenocarcinoma (MCF-7) and hepatocellular carcinoma (HepG2), obtained the Holding Company for Biological Products and Vaccines (VACSERA), Cairo, Egypt, on behalf of the American Type Culture Collection (ATCC). The culture medium used for the cells was RPMI-1640, supplemented at 37 °C in a humidified environment with 5% CO₂, with 10% fetal bovine serum (GIBCO, UK), 100U/mL penicillin, and 100 µg/mL streptomycin.

##### Reagents and standards

Quercetin, ferrous ammonium sulfate, and the iron(II)-quercetin complex were evaluated for cytotoxicity. Doxorubicin and sorafenib were used as standard reference drugs. The MTT reagent and DMSO were achieved from Sigma-Aldrich (USA).

##### MTT assay procedure

To ascertain cell viability, the MTT colorimetric test was utilized. Briefly, 1 × 10⁴ cells/well were scattered in 96-well plates and incubated for 48 h. After exposure to variable concentrations of the test compounds for 24 h, after adding 20 µL of MTT solution (5 mg/mL) to each well, the wells were protected for four further hours. The resulting formazan crystals were made soluble by adding 100 µL of DMSO, and a microplate reader (EXL 800, USA) was used to assess absorbance at 570 nm. Relative cell viability was calculated as:

$${{Viability (\% ) = (}}{{\mathrm{A}}_{{\mathrm{570}}}}{\text{ treated / }}{{\mathrm{A}}_{{\mathrm{570}}}}{\mathrm{control)}} \times {\text{100 }}$$

**Fig. 1:**
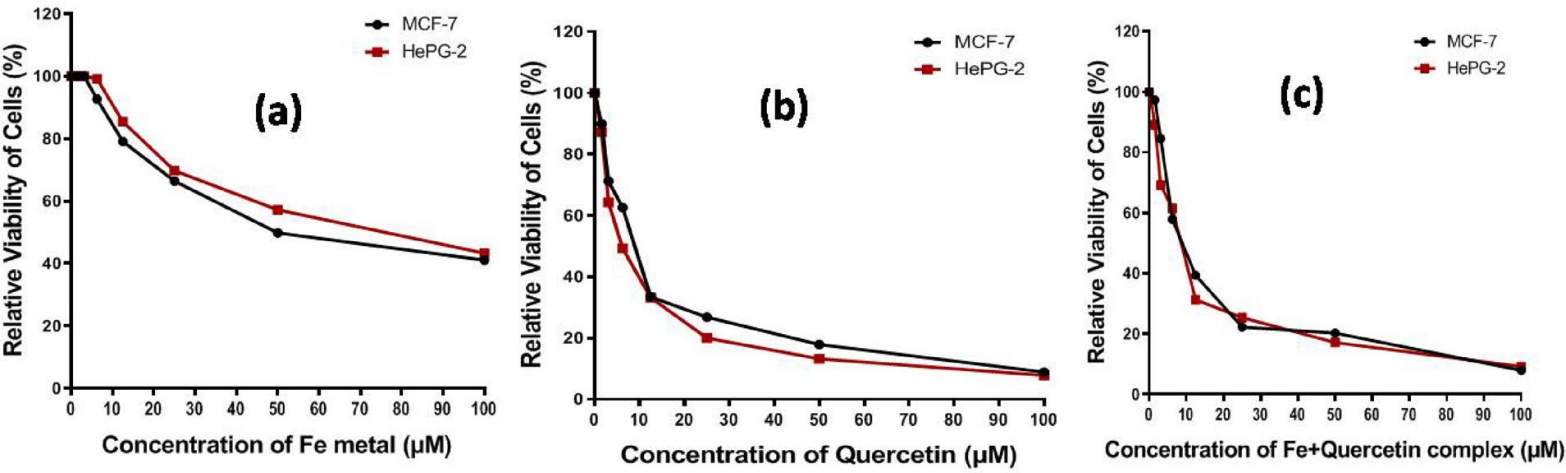
Viability of Two cell lines at various concentrations of (**a**) ferrous ammonium sulphate, (**b**)Quercetin and (**c**) Fe(II)-quercetin complex.

The values of IC₅₀ were determined from graphs of dose-response. According to the results, quercetinindicated the highest cytotoxicity compared to HepG2 cells (IC₅₀ = 8.14 µM), followed by the iron(II)-quercetin complex (26.80 µM). Against MCF-7 cells, IC₅₀ values were (9.75 µM) and (18.60 µM) for quercetin and the complex, respectively. Ferrous ammonium sulfate exhibited weak cytotoxic effects on both cell lines. The findings demonstrated a dose-dependent cytotoxic response, indicating enhanced bioactivity upon complexation (see in Fig. 1 and Table S1, S2). The data in (Table S1) represent cell viability (%) after treating two human cancer cell lines at different concentration for ferrous ammonium sulfate, quercetin and the iron (II)-quercetin complex, which shown in (Fig. 1) as indicating that DOX have very low viability (6–70%) even at low concentrations, so DOX have strong cytotoxicity due to Doxorubicin is a potent chemotherapy drug, SOR also strongly decreases viability, though slightly less than DOX. Ferrous Ammonium Sulphate shows high viability (40–100%) almost no cytotoxicity that confirms Fe²⁺ itself is not toxic at these levels and also quercetinshows moderate toxicity viability to around 8–60% depending on concentration. This confirms that quercetin alone has anticancer potential, but less than standard drugs. The viability data for Fe(II)-Quercetin complex shows dose-dependent reduction in cell viability. At 100 µM, (viability ≈ 8–9%) shows very strong effect (similar to DOX). At 25–12.5 µM, viability increases to (~ 22–40%) moderate toxicity. At 1.56 µM, viability is high (≈ 89–97%) very little effect. Finallythe Fe(II)-quercetin complex remains cytotoxic at higher doses, similar to free quercetin, but the pattern suggests slightly modified activity, likely due to the coordination of Fe(II) affecting cellular uptake or ROS generation.

#### Antioxidant activity assay by DPPH model

The antioxidant activity of quercetin, iron (II)-quercetin combination, and ferrous ammonium sulphate was assessed using a previously published technique with some changes employing the 2, 2-diphenyl-1-picrylhydrazyl (DPPH) radical scavenging assay^29^. Different concentrations of each compound were prepared and adjusted to a final volume of 3 mL with methanol. Subsequently, 1 mL of a 0.1 mM DPPH methanolic solution was added to each sample. The reaction mixtures were incubated in the dark at room temperature for 30 min.

A solution containing 1 mL of DPPH with concentration (0.1mM) and 3 mL of methanol was used as the control. The antioxidant standard, butylatedhydroxytoluene (BHT), was included for comparison.

The absorbance of each sample was determined at 517 nm with a UV-Vis spectrophotometer. The following formula was used to determine the percentage of radical scavenging activity:

$${\mathbf{Inhibition}}{\text{ }}\left( \% \right){\text{ }} = \left[ {\left( {{{\mathbf{A}}_{{\mathbf{control}}}}--{\text{ }}{{\mathbf{A}}_{{\mathbf{test}}}}} \right){\text{ }}/{{\mathbf{A}}_{{\mathbf{control}}}}} \right]{\text{ }}{\mathbf{x100}}$$

The absorbance of the test solution is denoted by A_test_ and the absorbance of the control by A_control_.The concentration of each compound required to inhibit 50% of the DPPH radicals (IC₅₀) was determined from a dose–response curve. All experiments were conducted in triplicate, and the mean ± standard deviation (SD) is used to display the IC₅₀ values.

The iron(II)-quercetin complex and free quercetin showed IC₅₀ values of 21.86 ± 0.16 µg/mL and 23.47 ± 0.15 µg/mL, respectively. Which indicate stronger antioxidant activity than ferrous ammonium sulfate, which had an IC₅₀ of 126.10 ± 0.67 µg/mL. The standard BHT exhibited the highest antioxidant activity with 16.81 ± 0.10 µg/mL as the IC₅₀ (Table 1). All results demonstrate the enhanced antioxidant potential of quercetin upon complexation with Fe(II) and indicating that, BHT (Standard) Expected to have the strongest radical scavenging ability because it’s a pure synthetic antioxidant designed for this function and its low IC₅₀ (16.81 µM) confirms high potency. The Fe (II)-Quercetin complex shows almost similar activity to BHT, even slightly better than quercetin alone, as complexation between Fe (II) and quercetin enhances electron transfer ability, improving the scavenging of free radicals and the Fe center may facilitate redox cycling, increasing reactivity with ROS (reactive oxygen species). Quercetin already a known strong antioxidant due to its phenolic –OH groups, which donate hydrogen atoms to neutralize free radicals. Slightly higher IC₅₀ than the Fe (II) complex meaning the complexation slightly improved antioxidant efficiency. Ferrous ammonium sulfate (Fe²⁺) Weak antioxidant (high IC₅₀ = 126 µM). In some cases, Fe²⁺ can even promote oxidation (via Fenton reaction), so it’s not an efficient radical scavenger alone^30^.

**Table 1:** Antioxidant activity of ferrous ammonium sulphate, Quercetin and Fe (II)-quercetin complex.

Compound	Conc. (µM)						
	10	20	40	60	80	100	IC50
	Inhibition (%)						
BHT	38.7	52.1	69.6	81.8	87.4	94.5	16.81 ± 0.10
Ferrous ammonium sulfate	8.1	15.9	23.7	30.5	38.2	46.6	126.10 ± 0.67
Fe(II)- Quercetin complex	30.4	45.8	62.3	76.8	89.3	96.1	21.86 ± 0.16
Quercetin	28.1	45.5	58.7	75.0	86.4	95.3	23.47 ± 0.15

#### Anti – microbial activity

The antibacterial and antifungal activity of the synthesized compounds—ferrous ammonium sulfate, quercetin, and the Fe(II)-quercetin complex—was evaluated against selected microbial strains, including the Gram-positive bacterium Staphylococcus aureus, the Gram-negative bacterium Escherichia coli, and the fungal strain Candida albicans. Dimethyl sulfoxide (DMSO) was used as a solvent to dissolve all compounds and prepare stock solutions at a concentration of 1 mg/mL. The antimicrobial activity was assessed using the agar diffusion method to determine zones of inhibition and evaluate the comparative efficacy of the compounds against bacterial and fungal pathogens. Sterile paper discs (5 mm diameter, Whatman filter paper) were prepared and impregnated with the test solutions. The discs were then aseptically placed on nutrient agar plates (composition: Peptone 5 g, beef excerpt 3 g, and agar 20 g per liter) inoculated with the target microorganisms. The plates were nurtured at 36 °C for 24 h, and the regions of inhibition were millimeters in size. A triple of each test was conducted.

Ciprofloxacin and clotrimazole were expended as reference criteria for the antifungal and antibacterial properties, respectively, with the same level of concentration and under identical conditions. Each test compound’s Activity Index (%) was determined using the formula below^30^:

$$\% \;Activity\;Index = \;\frac{{Zone\;of\;inhibition\;by\;test\;compound\;\left( {diametre} \right)}}{{Zone\;of\;inhibition\;by\;standard\;\left( {diametre} \right)}}\times100$$

The results (Table 2) demonstrated that all three compounds exhibited measurable antimicrobial activity. Statistical analysis using one-way ANOVA revealed significant differences in inhibition zones among the tested compounds (*p* = 0.00). Against E. coli, quercetin and the Fe (II)-quercetin complex showed moderate activity, producing inhibition zones of 12 mm and 10 mm, respectively, while ferrous ammonium sulfate exhibited no measurable inhibition. For S. aureus, both quercetin (14 mm) and the Fe (II)-quercetin complex (11 mm) demonstrated notable antibacterial activity compared with the standard drug ciprofloxacin (24 mm). In the case of Candida albicans, the Fe(II)-quercetin complex and quercetin exhibited inhibition zones of 15 mm and 19 mm, respectively, relative to the reference antifungal clotrimazole (27 mm). Overall, these findings confirm that quercetin and its Fe (II) complex possess significant antimicrobial properties, with complexation enhancing quercetin’s effectiveness in certain cases.

**Table 2:** Antimicrobial activity of ferrous ammonium sulphate, Quercetin and Fe (II)-quercetin complex.

Compound	E. coli		S. aureus		C. Albicans	
	Diameter of inhibition zone (mm)	% Activity index	Diameter of inhibition zone (mm)	% Activity index	Diameter of inhibition zone (mm)	% Activity index
Ferrous ammonium sulfate	NA	----	NA	----	4	14.8
Fe(II)-Quercetin complex	10	38.5	11	45.8	15	55.5
Quercetin	12	46.1	14	58.3	19	70.4
Ciprofloxacin	26	100	24	100	----	----
Colitrimazole	----	----	----	----	27	100

NA → No Activity.

**----** → Noapplicable for that organism or condition.

### Molecular docking

Simulations of molecular docking were carried out utilizing the **Molecular Operating Environment (MOE) 2015** software to evaluate the binding affinity of quercetin and its iron(II) complex with the SARS-CoV-2 spike protein (PDB ID: 7JWY). The protein structure was retrieved from the Protein Data Bank and prepared for docking by removing water molecules and adding missing hydrogen atoms.

The ligands (quercetin and iron(II)-quercetin complex) were sketched using **ChemDraw 15.0**, and their energy was minimized using the **MMFF94x** force field in both gas and solvent phases to ensure stable conformations. Protonation was performed to add explicit hydrogen atoms, especially those absent in crystal structures.

The identification of the protein’s active location using the **Site Finder** module in MOE, and key interacting residues were analyzed. Molecular docking was then carried out using the **Dock module**, with the **London dG** scoring function selected for ranking binding poses. The docking protocol generated 30 poses per ligand, and the receptor was kept rigid during simulations. The final docked complexes were analyzed for binding energy and interaction profiles.

For COVID-19 molecular docking investigations, the 7JWY protein was chosen based on a number of important considerations:

- Biological Significance: SARS-CoV-2 is the virus that is causing the COVID-19 pandemic. Understanding the interactions between viral proteins and potential drugs is crucial for the creation of novel therapeutics.
- Spike Protein Targeting The 7JWY protein has a specific structure that corresponds to the SARS-CoV-2 spike protein. The spike protein’s receptor-binding domain (RBD) is essential for the virus’s entry into host cells. The RBD can be inhibited to disrupt viral infection.
- Accessibility of Structural Data: The 7JWY protein structure is readily available in the Protein Data Bank (PDB) and has undergone extensive characterization, making it a strong contender for molecular docking studies.
- The 7JWY protein is a promising target for molecular docking studies to find possible therapeutic targets.
- Previous Research: Prior studies or articles have demonstrated the significance of focusing on the 7JWY protein when creating novel medications or comprehending the mode of action of possible antiviral substances^30,31^.

Despite being very useful and instructive, molecular docking studies have a number of drawbacks^32,33^. Scoring Functions: Approximations of the true binding energies are used in the docking process as scoring functions. There may not always be a strong association between them and experimental affinities & Binding Kinetics: Affinity, or binding thermodynamics, is the primary field of research for docking. The rates of attachment and dissociation, as well as kinetics, are not specifically discussed.

Quercetin and iron (II)-quercetin complex molecular docking with a target protein SARS-CoV-2 improves our knowledge of the potential ways in which the two drugs interact and bind to the protein receptors, which can provide further information about the compounds’ capacity to exert bioactivity towards the target protein^32,33^. To find out how successfully the investigated compounds dock with the SARS-CoV-2 protein, score grades and docking interaction energy are calculated^34,35^. Complex quercetin’sstructure has been investigated^35^.

 The docking results revealed a total binding energy of − 5.425 kcal/mol for quercetin and − 4.732 kcal/mol for the Fe(II)–quercetin complex (Table S3). The primary interacting residues within the 7JWY protein included Glu227, Gly339, Ser371, and Pro, which play crucial roles in ligand binding and catalytic activity. Additional residues—Met, Phe, Val, Leu, Lys, Ile, and Thr—were also involved in maintaining the structural stability of the protein. Quercetin was found to bind within the receptor pocket through a hydrogen bond between its O22 atom and Ser371, while the Fe(II)–quercetin complex formed hydrogen bonds between its O20 and O22 atoms and the residues Gly1131 and Thr1077, respectively.

**Fig. 2:**
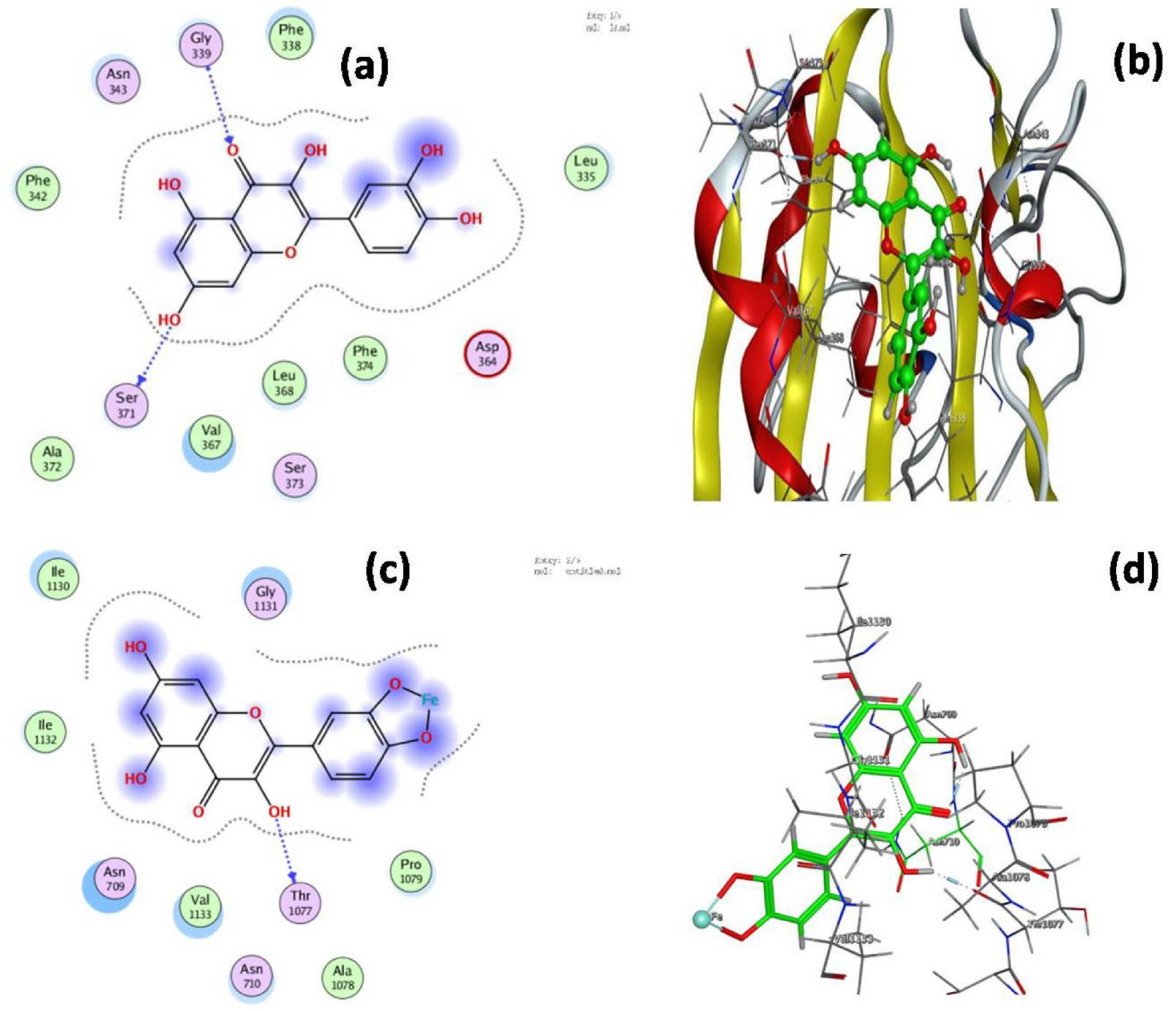
Interaction diagram showing quercetin and iron (II)-quercetin complex docking interactions with7JWY protein (**a**) The 2D interaction of quercetin with ACE2 receptor of SARS-CoV-2.(**b**) The 3D interaction of quercetin with ACE2 receptor of SARS-CoV-2. (**c**) The 2D interaction of complex with ACE2 receptor of SARS-CoV-2. (**d**) The 3D interaction of complex with ACE2 receptor of SARS-CoV-2.

Glu227, Gly339, Ser371, and Pro are amino acid residues that are known to be crucial for interactions and are often found in the active portion of the 7JWY protein. The formation of hydrogen bonds and hydrophobic interactions between these residues and ligands is essential for the protease’s enzymatic activity and contributes to catalysis and substrate recognition. Methionine (Met), phenylalanine (Phe), valine (Val), leucine (Leu), lysine (Lys), isoleucine (Ile), and threonine (Thr) are among the other frequent amino acids that may be present in the 7JWY protein structure, and also might support the conservation the stability and structure of protein, (Figs. 2a, b) show that these atoms might be required for the inhibitor complexation phase. The location where quercetin attaches itself to the protein receptor pocket using a ligand-active H hydrogen bond, Ser371. The inhibitor interactions in 7JWY docking with the iron (II)-quercetin complex through O 20 and O 22 atoms towards the protein receptor pockets Gly1131 and Thr1077 were found to be validated by the presence of a broad attractive region near the residues Gly339, Ser371, and Asn343, as illustrated in (Figs. 2c, d). The MOE docking reports indicated that the root mean square deviation (RMSD) values for quercetin and the Fe (II)–quercetin complex were 5.424 Å and 4.737 Å, respectively, confirming comparable docking conformations and stable interactions. These findings suggest that both ligands interact effectively with the spike protein’s receptor-binding domain, highlighting their potential as lead compounds for antiviral drug design against SARS-CoV-2.

Using molecular docking studies to understand the active sites of the 7JWY protein and how they interact with potential inhibitors will provide valuable insights for COVID-19 drug discovery efforts. To validate these findings and evaluate their efficacy in treating COVID-19, however, more experimental studies are needed.

### UV–visible spectral determination of Quercetin and its iron(II) complex (with continuous variation method)

**Fig. 3:**
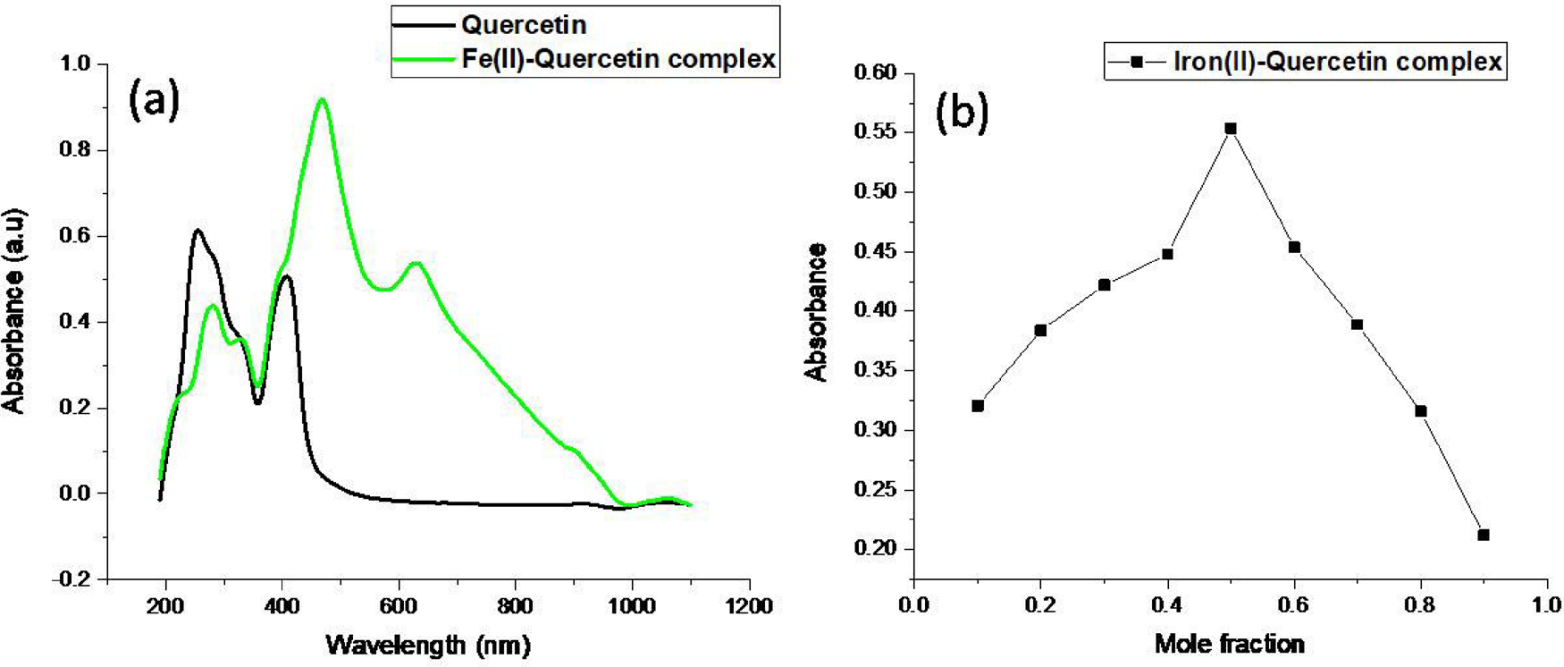
(**a**) UV-visible spectroscopy for quercetin and iron (II)-quercetin complex. (**b**) Continuous variation between ferrous ammonium sulphate and quercetin.

Spectrophotometric analysis of quercetin and the iron(II)-quercetin complex using UV–visible spectroscopy was performed in ethanol over the range of 200–600 nm to investigate the electronic transitions and confirm complex formation. Quercetin exhibited two characteristic absorption bands: Band II appeared around 256 nm, attributed to π→π* transitions in the benzoyl structure of the A-ring, and Band I near 372 nm, corresponding to n→π* transitions in the cinnamoyl structure of the B-ring. These are typical features of flavonoids. Upon coordination with iron (II), the spectrum showed notable bathochromic shifts and new absorption peaks at approximately 273 nm and 438 nm. These shifts indicate electronic reorganization caused by ligand-to-metal charge transfer. The appearance of these new peaks with enhanced absorbance intensity confirms the formation of a stable iron(II)-quercetin complex involving coordination between Fe(II) ions and the hydroxyl and/or carbonyl groups of quercetin, as shown in (Fig. 3a).To further determine the stoichiometry of the complex the continuous variation method was applied by preparing equimolar solutions of ammonium ferrous sulphate and quercetin each at 5 × 10⁻³ M concentration in distilled water and mixing them in varying volume ratios ranging from 1:30 to 30:1 while conserving a constant total volume of 1 L and the absorbance of each mixture was measured at the λ_max_ of 468 nm and from the plot generated in (Fig. 3b) the maximum absorbance was observed at a molar fraction of 0.5 indicating that the optimal ratio between metal and ligand is 1:1 which strongly provisions the formation of a 1:1 stoichiometric complex between quercetin and Fe(II).

### ¹H nmrspectral determination of Quercetin and its iron (II) complex

¹H NMR (proton nuclear magnetic resonance) spectrum of quercetin and Fe (II)-quercetin complex, showed in (Fig. S2),¹H NMR for quercetin, in (Fig. S2a) indicates that there are peaks based on the ppm values at 12.49, 10.80 and 9.61–9.33 represent OH-phenolic group, where at 12.49 ppm hydrogen-bonded hydroxyl group (common in flavonoids), at 10.80 ppm a strongly de shielded due to¹H NMR conjugation and H-bonding and a range from 9.61to 9.33 de shielded more hydroxyl protons, possibly intra-molecularly H-bonded. Finally, the range from 7.66 to 7.51 indicates aromatic protons from B-ring of quercetin (meta/ortho coupled doublets). Quercetin is a flavonol with five hydroxyl groups, its structure consists of two benzene rings (A and B), a central heterocyclic pyrone ring (C) and Hydroxyls at positions: 3, 5, 7, 3′, and 4′ each hydroxyl group gives broad singlets at downfield regions (9–13 ppm), while aromatic protons appear as multiplets or doublets between 6 and 8 ppm, depending on the ring they are on and coupling with neighboring protons^9,36–40^, and also proton NMR for Fe (II)-quercetin complex in (Fig. S2 b) illustrated that some phenolic OH protons are slightly shifted from 9.61 to 9.63 ppm, which indicates Fe (II) coordination through oxygen atoms most likely at 3-OH and 4-carbonyl or 5-OH and 4-carbonyl, common coordination sites for flavonols, and also aromatic protons moved slightly, as suggesting electron density changes due to Fe binding, where quercetin’s conjugated π-system interacts with Fe²⁺ which affecting shielding of nearby protons. On the other hand, the Fe (II)-quercetin complex is stable in solution, coordination likely involves hydroxyl and carbonyl groups, especially at C3, C4, and possibly C5 and downfield shifts confirm metal-ligand interaction^41–45^.

### X-ray diffractionof Quercetin and its iron (II) complex

**Fig. 4:**
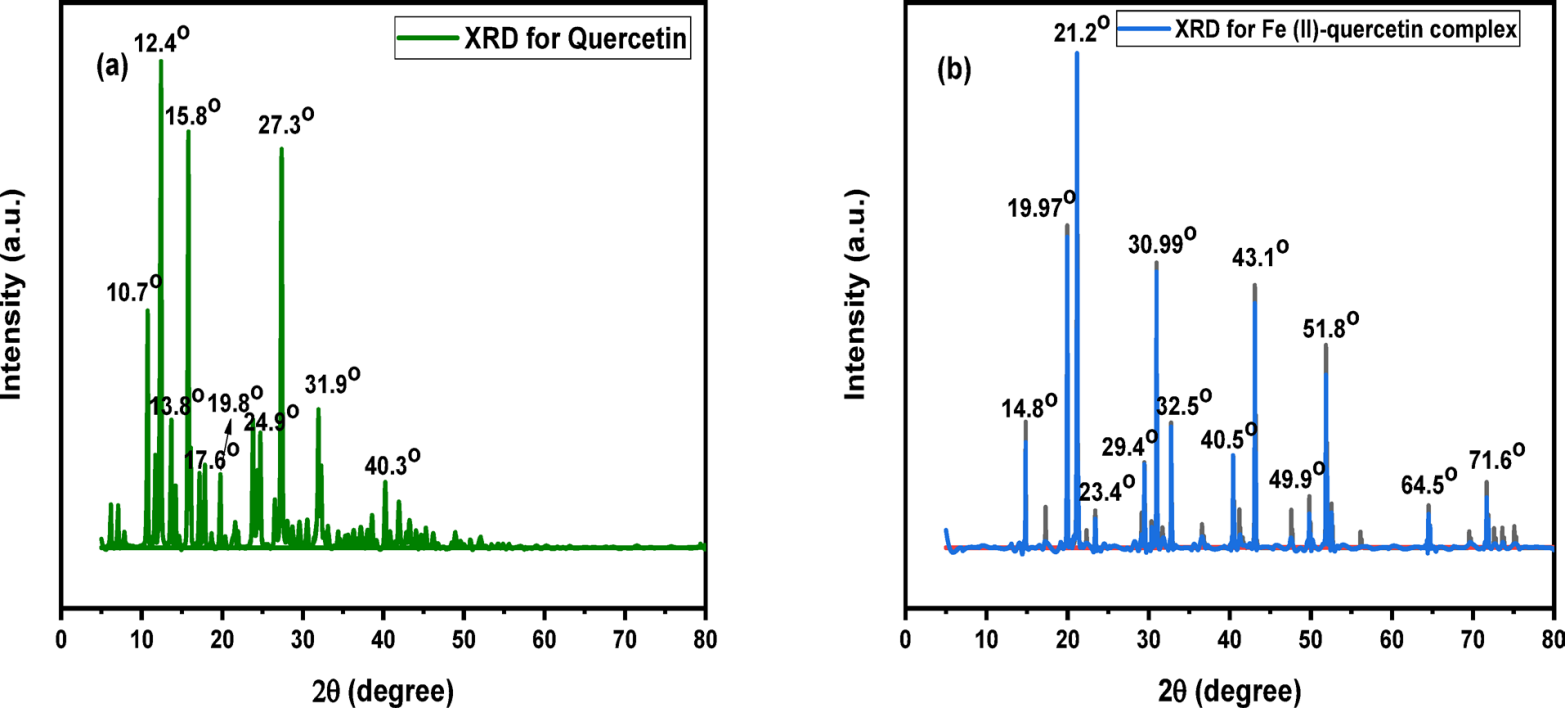
XRD patterns for (**a**) Quercetin and (**b**) Fe (II) - quercetin complex.

The XRD **(X-ray Diffraction)** identifies the crystalline phases in a material and gives insight into: **Layer spacing (basal spacing)** in quercetin, showed in (Fig.4a) a sharp peaks at 2 θ (10.7°, 12.4°, 13.8°, 15.8°, 17.6°, 19.8°, 24.9°, 27.3°, 31.9°, 40.3°) indicate a crystalline structure. Peaks around 10–20° are characteristic of intermolecular stacking and lattice periodicity, which are typical for small organic crystals like quercetin. The peak at ~ 24.9° may be attributed to π–π stacking between aromatic rings, which is common in flavonoid crystals and the peaks above 30° suggest more compact and possibly ordered packing in certain crystallographic planes. In (Fig. 4b) for Fe (II)-quercetin complex, peak positions (2θ in degrees): 14.8°, 19.97°, 21.2°, 23.4°, 29.4°, 30.99°, 32.5°, 40.5°, 43.1°, 49.9°, 51.8°, 64.5°, 71.6° analysis, which compared to pure quercetin. Each peak corresponds to diffraction from specific lattice planes in the crystalline material, as per Bragg’s Law (n λ = 2dsinθ) and give (100), (110), (111), (200), (210), (211), (221), (310), (311), (400) and (411). This pattern, shows new peaks (e.g., 29.4°, 30.99°, 32.5°, 43.1°), which are not present in the quercetin curve, suggesting formation of a new crystalline phase confirming complexation and also, some original quercetin peaks disappear or shift, indicating that coordination with Fe(II) alters the molecular packing. The increased number of peaks and slight broadening implies reduced crystallinity or different crystal symmetry. These changes reflect alterations in crystal structure and symmetry due to Fe (II) binding, likely involving chelation through hydroxyl and/or carbonyl groups of quercetin arrangement different from pure quercetin. The structural rearrangement likely involves chelation of Fe (II) at key sites on the quercetin molecule, forming a stable complex with modified packing and lattice symmetry. Finally, the shift in peak positions and appearance/disappearance of certain peaks implies, formation of a new coordination compound, change in molecular packing and interplanar spacing and possible decrease in crystallinity or formation of an amorphous phase alongside crystalline components^45^.

## Result and discussion

### Theoretical information about Cyclic voltammetry

Ferrous ammonium sulphate and quercetin’s electrochemical behavior and interaction mechanism will be examined. First, ion-stable condition curves were used to examine the electrochemical redox behavior of quercetin, iron (II), and their complex. Equation 1 is used to determine the diffusion coefficient and to detect and describe cyclic waves because it provides information on how the current reacts to changes in concentration and explains how the peak current increases linearly with the square root of the scan rate^46^.

1 $$\:{\mathbf{i}}_{\mathbf{p}}=0.4463\:\mathbf{n}\:\mathbf{F}\:\mathbf{C}\:\mathbf{A}\:{\left(\frac{\mathbf{n}\:\mathbf{F}\:\mathbf{D}\:\boldsymbol{\upnu\:}}{\mathbf{R}\:\mathbf{T}}\right)}^{\raisebox{1ex}{$1$}\!\left/\:\!\raisebox{-1ex}{$2$}\right.}$$

A is the surface area of the working electrode in cm^2^, v is the scan rate in volts/sec, i_p_is the current in amps, C is the concentration, and D is the diffusion coefficient in cm^2^/sec. Additionally, Eq. 2 is used to determine ΔE_P_^47^..

2 $$\:{\boldsymbol{\Delta\:}\mathbf{E}}_{\mathbf{p}}={-\mathbf{E}}_{\mathbf{p}\mathbf{c}}+\:{\mathbf{E}}_{\mathbf{p}\mathbf{a}}$$

The typical heterogeneous electron transfer rate constant, k_s_, in cm/s was determined using Eq. 3^48^.

3 $$\:{\mathbf{k}}_{\mathbf{s}}=2.18\:{\left(\frac{{\mathbf{D}}_{\mathbf{c}}{\mathbf{n}}_{\mathbf{a}}\:\mathbf{F}\:\boldsymbol{\upnu\:}\:\boldsymbol{\upalpha\:}}{\mathbf{R}\mathbf{T}}\right)}^{\raisebox{1ex}{$1$}\!\left/\:\!\raisebox{-1ex}{$2$}\right.}\mathrm{*}\mathbf{exp}\left(\:\:\frac{\mathbf{n}\:\mathbf{F}\:{\boldsymbol{\upalpha\:}}^{2}{\boldsymbol{\Delta\:}\mathbf{E}}_{\mathbf{p}}}{\mathbf{R}\mathbf{T}}\:\right)\:\:\:\:\:\:\:\:\:$$

Where the number of electrons that move during the rate-determining phase is denoted by n_a_. Given that the coefficient has a value of 0.5, Eq. 4 will therefore be used to calculate n_a_^49^.

4 $$\:{\boldsymbol{\upalpha\:}}_{{\mathbf{n}}_{\mathbf{a}}}=\left(\frac{1.857\:\mathbf{R}\mathbf{T}}{\left({\mathbf{E}}_{\mathbf{p}\mathbf{c}\:}-\:{\mathbf{E}}_{\mathbf{p}\mathbf{c}/2}\right)\:\mathbf{F}}\right)$$

The cathodic peak half-wave potential is represented by the symbol E_pc_. Next, Eq. 5 was used to evaluate the surface coverage^50^.

5 $$\:\boldsymbol{\Gamma\:}=\:\frac{{4\:\mathbf{T}\:\mathbf{R}\:\mathbf{i}}_{\mathbf{p}}}{{\mathbf{n}}^{2}{\mathbf{F}}^{2}\:\boldsymbol{\upnu\:}\:\mathbf{A}}$$

Surface coverage is calculated using Eq. 6 and the amount of charge consumed in the adsorption or decrease of the adsorbed layer^51^.

6 $$\:\boldsymbol{\Gamma\:}=\:\frac{\mathbf{Q}}{\mathbf{n}\:\mathbf{F}\:\mathbf{A}}$$

Additionally, Eq. 7 can be used to determine the amount of charge consumed when the adsorbed layer on the working electrode is reduced, oxidized, or adsorbed based on the surface coverage concentration of the layer.

7 $$\:\mathbf{Q}=\mathbf{n}\:\mathbf{A}\:\boldsymbol{\Gamma\:}\:\mathbf{F}$$

The stability constant (β_MX_) for complexes calculated using Eq. 8 measures the degree of interaction among the ingredients that join together to form the complex^52,53^.

8 $$\:\varDelta\:\mathbf{E}^\circ\:=2.303\left(\frac{\mathbf{R}\mathbf{T}}{\mathbf{n}\mathbf{F}}\right)\left(\:\mathbf{j}\:\mathbf{l}\mathbf{o}\mathbf{g}{\mathbf{C}}_{\mathbf{X}}\:+\:\mathbf{l}\mathbf{o}\mathbf{g}{\boldsymbol{\upbeta\:}}_{\mathbf{M}\mathbf{X}}\right)={\mathbf{E}^\circ\:}_{\mathbf{C}}-\:{\mathbf{E}^\circ\:}_{\mathbf{M}}$$

Where T is the absolute temperature, C_X_ is the ligand concentration in the solution, E^o^_C_ is the formal peak potential of the metal complex after each addition of quercetin, The E^o^_M_ is the metal’s formal peak potential at the final addition, when no ligand is present, and j is the coordination number of the stoichiometric complex. Using Eq. 9, the formal potentialE^o^ can be found.

9 $$\:\mathbf{E}^\circ\:=\left(\frac{{\mathbf{E}}_{\mathbf{P}\mathbf{c}}+\:{\mathbf{E}}_{\mathbf{P}\mathbf{a}}}{2}\right)$$

E_pc_ has a cathodic peak potential, while E_p.a._ has an anodic peak potential. The Gibbs free energy of the complexation between quercetin and iron (II) can be found using Eq. 10 and the stability constant (β_MX_).

10 $$\:-\varDelta\:\mathbf{G}=\left(2.303\right)\mathbf{R}\mathbf{T}\mathbf{log}{\boldsymbol{\upbeta\:}}_{\mathbf{M}\mathbf{X}}$$

### The electrochemical behavior study of Quercetin

Using a glassy carbon electrode and cyclic voltammetry, the oxidation behavior of quercetin was investigated at 303.05 K with 0.1 M potassium chloride as a supporting electrolyte and ethanol as a solvent. The open window circuit extended between 1.5 and − 1.5 V, and the scan rate is 0.1 V/s. Quercetin is added with concentration (0.01 M) it^’^s cyclic voltammetry as shown in (Fig.S3).The lack of observable redox peaks in the CV curve supports the hypothesis that quercetin may act primarily through these radical-scavenging mechanisms rather than undergoing direct electrochemical oxidation or reduction, which studied from antioxidants can exert their protective effects through two primary pathways, both of which have been extensively studied and proposed^16–18,54^. The conversion of hydrogen atoms from the antioxidant (ArOH) to a free radical (R^•^):

$${R^ \bullet } + {\text{ }}ArOH \to RH{\text{ }} + {\text{ }}Ar{O^ \bullet }{\mathbf{x100}}$$

$${R^ \bullet } + {\text{ }}ArOH \to {R^ - } + {\text{ }}ArO{H^{ \bullet + }}$$

**Fig. 5:**
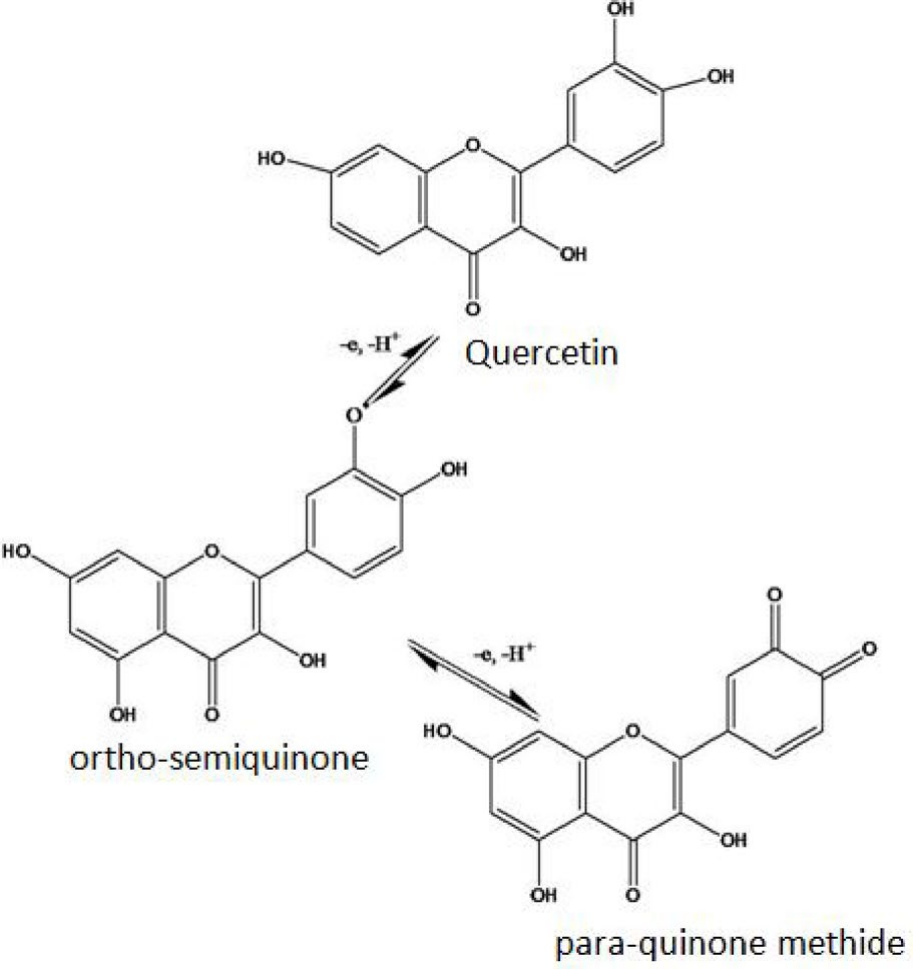
Electro-oxidation of quercetin.

The mechanism of one-electron transfer, according to which the free radical can receive an electron from the antioxidant:

One-electron and one-proton participation in the oxidation of quercetin results in the synthesis of ortho-semiquinone, which can then undergo additional oxidation to generate the para-quinonemethide, as shown in (Fig. 5).

### The electrochemical behavior study of the freeferrousionsat 303.05 K

**Fig. 6:**
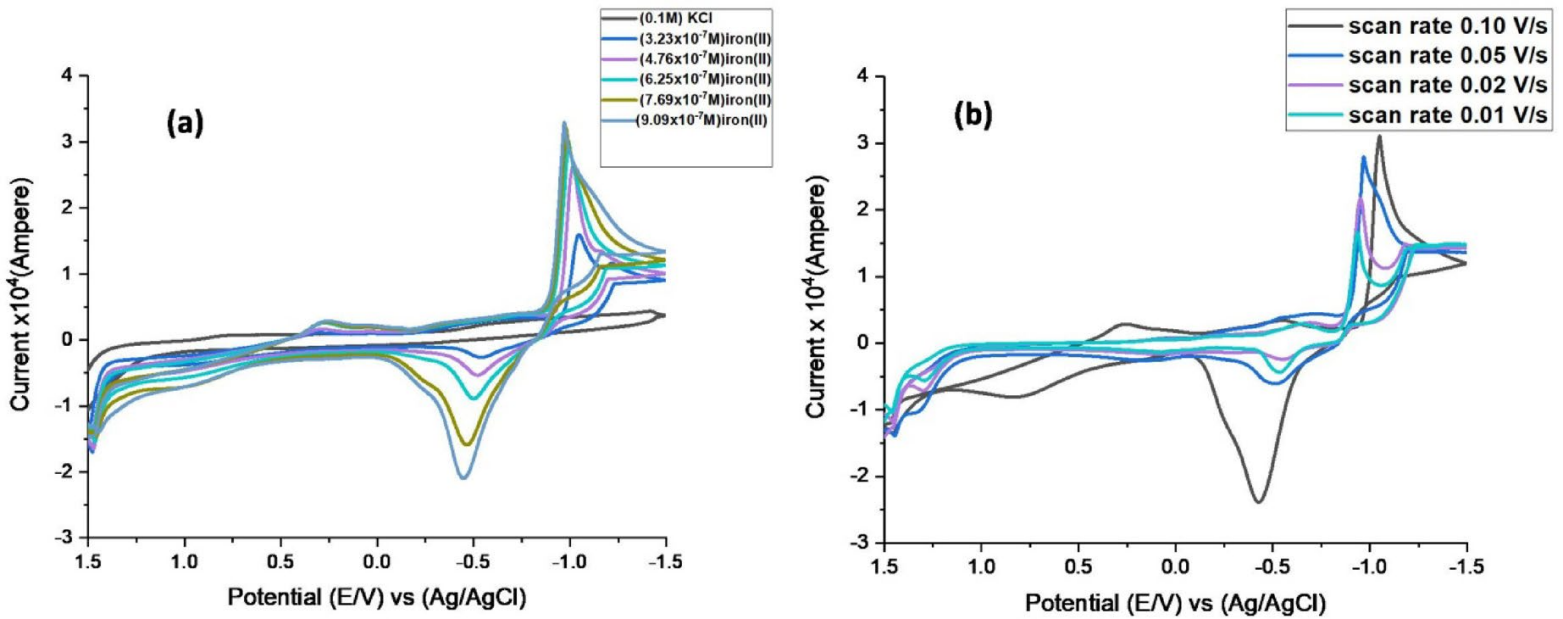
(**a**) Electrochemical behavior of iron (II) with Different concentration (scan rate 0.1 V/s) at strong electrolyte KCl (0.1M)(**b**)Electro-chemical behavior of iron (II) at different scan rate.

The oxidation and reduction behavior of ferrous ammonium sulfate was scanned in 0.1 M KCl as a supporting electrolyte.Various concentrations of (NH_4_)_2_SO_4_.FeSO_4_.6H_2_O were used, ranging from 3.23 × 10^− 7^M to 9.09 × 10^− 7^M and at a temperature of 303.05 K. From1.5 to −1.5 V was the measurement range of the potential window. Display one oxidation peak and one reduction peak, as seen in (Fig. 6a), as the concentration of iron (II) increased, so did the reduction and oxidation peak current (I_p_). The sharpened oxidation and reduction peak was seen at roughly − 0.45 and − 0.97 V.Explain that redox reaction of Fe^2+^/Fe^3+^ through the mechanism shown in Eq. 11.

The Ferrous ammonium sulphatebehavior was also revised at different scan rates, as exhibited in (Fig. 6b). Scanned cyclic voltammograms have been used to study conjugation’s reversibility, and many distinct data points are displayed on E_pc_, E_pc/2_, I_pc_, I_p.a._, and E_p.a._. These collected characteristic information might be used to determine the difference potential (∆E_p_ = - (E_pc_) + E_p.a._), the number of electron transfers in the rate-determining step (na), and the current ratio (I_p.a._/I_pc_), as indicated in (Tables 3 and 4). Cyclic voltammetry (CV) analysis plot, peak current (ip) is the maximum current measured during the redox process. Scan rate (ν) is the rate at which the potential (voltage) is swept in the CV experiment (in V/s). The square root of the scan rate (v^1/2^) is used because, for a diffusion-controlled process, the Randles–Ševčík equation predicts a linear relationship between ip and v^1/2^. Showed in (Fig, S4), which demonstrates that the peak current of ferrous ammonium sulfate increases linearly with the square root of the scan rate, indicating a diffusion-controlled redox process at 303.05 K.

11 $$F{e^{2 + }} \rightleftharpoons F{e^{3 + }}{e^ - }$$

Q and Γ values were determined for the reduction and oxidation processes at each scan rate, as indicated in (Table S4). As the redox process time increases, the concentrations of surface coverage at the glassy carbon electrode surface increase as the scan rate decreases. This implies that the solution and the surface of the glassy carbon electrode are also exchanging an increasing amount of charge.

**Table 3:** Study of different concentration of ammonium ferrous sulphate by using glassy carbon electrode at 303.05 K and scan rate 0.1 V.S^− 1^.

[M] X10^7^ Mol.L^− 1^	Ep, a volt	Ep, c volt	∆Ep volt	(-)Ip, a X10^4^ Amp	Ip, c X10^4^ Amp	Ip, a/Ip, c
3.23	−0.547	−1.046	0.499	−0.323	0.932	−0.3467
4.76	−0.522	**-**1.015	0.493	−0.616	2.09	−0.2948
6.25	−0.503	−0.994	0.491	−1.08	2.38	−0.4533
7.69	−0.464	−0.980	0.516	−1.79	2.62	−0.6834
9.09	−0.449	−0.968	0.519	−2.32	2.75	−0.8461
**[M]** **X10** ^**7**^ **Mol.L** ^**− 1**^	**E˚** **volt**	**Da** **X10** ^**5**^ **Cm**^**2**^.**s**^**− 1**^	**Dc** **X10** ^**4**^ **Cm**^**2**^.**s**^**− 1**^	**Epc/2**	**αnac**	**Ks**
3.23	−0.7965	1.406	1.17	−1.013	1.469	6.6427
4.76	−0.7685	0.458	2.70	−0.98	1.386	9.2451
6.25	−0.7485	0.820	2.04	−0.961	1.469	8.1267
7.69	−0.722	1.488	1.63	−0.951	1.672	9.8404
9.09	−0.7085	1.790	1.28	−0.935	1.469	8.4112

**Table 4:** Study of different scan rates on the Cyclic voltammetry of ammonium ferrous sulphate.

Scan rate	Ep, a volt	Ep, c volt	∆Ep volt	(-)Ip, a X10^4^ Amp	Ip, c X10^4^ Amp	Ip, a/Ip, c
0.10	−0.428	−1.049	0.621	2.280	1.60	0.867
0.05	−0.515	**-**0.970	0.455	0.725	0.401	0.342
0.02	−0.551	−0.950	0.399	0.556	0.560	0.365
0.01	−0.531	−0.937	0.406	0.398	0.655	0.338
**Scan rate**	**E˚** **Volt**	**Da** **X10** ^**9**^ **Cm**^**2**^.**s**^**− 1**^	**Dc** **X10** ^**8**^ **Cm**^**2**^.**s**^**− 1**^	**Epc/2**	**αnac**	**Ksx10** ^**1**^
0.10	−0.739	8.830	1.175	−1.015	1.426	2.11
0.05	−0.743	1.178	1.527	−0.940	1.616	0.369
0.02	−0.751	2.633	1.967	−0.921	1.672	0.158
0.01	−0.734	2.690	2.362	−0.908	1.672	0.131

### The study of electrochemical complexation of iron (II) and Quercetin

**Fig. 7:**
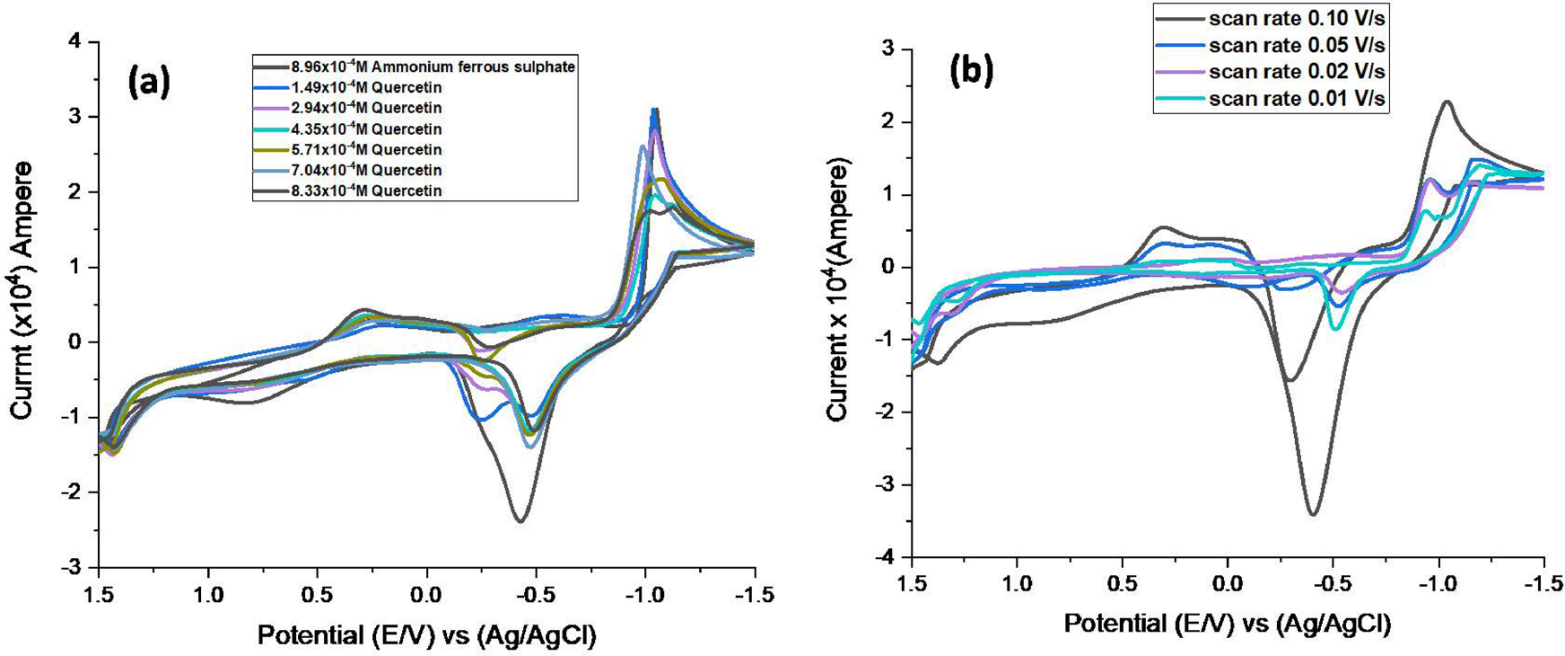
(**a**) Effect of different concentration of quercetin on ferrous ions at 303.05 K and scan rate 0.1 V/s. (**b**) Effect of varying scan rates on the cyclic voltammetry of ferrous ions with quercetin at 303.05 K.

 The electrochemical behavior of the ferrous ions and quercetin interaction was investigated using a glassy carbon electrode and cyclic voltammetry at a scan rate of 0.1 V/s in 0.1 M KCl as a supporting electrolyte at a constant temperature of 303.05 K, asilluminatedin (Fig. 7a). The complex was produced as a result of a significant shift in the cathodic potential towards new values, as well as a decline in the cathodic and anodic peaks, as seen in (Tables 5 and 6). This change indicates the reaction of complex formation^55^. Also, changes in the peak currents and positions in the CV curves can indicate how quercetin affects the redox behavior of ferrous ions. As the concentration of quercetin increases, you may observe variations in the peak current and potential. Higher concentrations of quercetin can enhance the electron transfer process, leading to increased peak currents. This suggests that quercetin may facilitate the oxidation of Fe^2+^ ions or stabilize the resulting products.This study shows clear electrochemical evidence of interaction between iron (II) and quercetin. Complexation leads to observable shifts in redox peaks during cyclic voltammetry, which is consistent with the formation of a coordination complex. These findings help understand how quercetin might interact with metal ions in biological or environmental settings, another piece of evidence for complex formation, additionally, as illuminated in (Fig. 7b).Experiments were performed at constant concentrations of 8.57 × 10^−4^M and at different scan rates. The complexation interaction causes an increase in the negative shifts (Epc) of the Fe^2+^ ions. Moreover, the ∆Ep values are higher than the free Fe^2+^ ion values. Consequently, adding the ligand to the Fe^2+^ ion solution reduces the reversibility of the process.Using the continuous variation, the charge transfer coefficient value was utilized to determine the kind of complexation mechanisms for the iron and quercetin peaks, as shown in (Figs. 7). Iron and quercetin were shown to react by two distinct mechanisms. The first was a quasi-reversible reaction, which was caused by the charge transfer coefficients for all scan speeds. The second was an irreversible reaction with quercetin and Fe^2+^. The oxidation and reduction peaks vanished, causing the charge transfer coefficients to drop until they were zero.The values of E^°^, β_MX_, and ΔG for the quercetin-iron complex are registered, both estimated and compiled. D, k_S_, Γ, and Q the kinetic and solvation parameters for the ferrous-quercetin complex were calculated and are shown in (Table S5). The cyclic voltammetry data support the formation of the Fe(II)–quercetin complex. The noticeable negative shift in the reduction potential (Epc) of Fe²⁺ and the significantly larger ∆Ep values compared with those of free Fe²⁺ ions indicate strong complexation between quercetin and iron (II). This complexation alters the electrochemical behavior of the system by modifying the redox kinetics, thereby confirming the formation of the iron(II)–quercetin complex. This might be reflected in the shifts of peak potentials or changes in the reversibility of the electrochemical reactions.The results can provide comprehensions into the thermodynamic stability of the iron (II) -quercetincomplex. If the electrochemical response shows significant changes with increased quercetin concentration, it suggests stronger binding and stabilization of the ferrous ions in the complex.

Linear relationship between peak current (Ip) and √scan rate (v^1/2^): Suggests that the redox process is diffusion-controlled. According to the Randles–Ševčík equation for reversible systems:

$$Ip = {\text{ }}\left( {{\text{ }}2.69{\text{ }} \times {\text{ }}{{10}^5}} \right) \cdot {n^{3{\text{ }}/{\text{ }}2}} \cdot A \cdot {D^{1{\text{ }}/{\text{ }}2}} \cdot C \cdot {v^{1{\text{ }}/{\text{ }}2}}$$

Where: : peak current (A), *n*: number of electrons transferred, *A*: electrode area (cm²), *D*: diffusion coefficient (cm²/s), *C*: concentration (mol/cm³), *ν*: scan rate (V/s). In (Fig. S5) showed linearity, which confirms that the electron transfer is governed by diffusion of quercetin or the complex to the electrode surface. This behavior is typical for small molecules undergoing redox reactions in solution.

The calculated electrochemical parameters (D, ks, and α) are summarized in (Table 7), confirming a decrease in ks and increase in ΔEp upon complex formation.

**Table 5:** Cyclic voltammetric data of Quercetin with ferrous ions in supporting electrolyte of KCl (0.1 M) at constant scan rate 0.1 V/s.

[M] X10^− 4^ Mol.L^− 1^	[L] X10^− 4^ Mol.L^− 1^	Ep, a Volt	Ep, c volt	∆Ep volt	(-)Ip, a X10^− 4^ Amp	Ip, c X10^− 4^ Amp	Ip, a/Ip, c
8.96	1.49	−0.476	−1.034	0.558	0.766	2.49	0.3074
8.82	2.94	−0.472	−1.046	0.574	1.10	2.18	0.5035
8.70	4.35	−0.475	−1.043	0.568	1.14	1.40	0.8174
8.57	5.71	−0.470	−1.061	0.591	1.23	1.52	0.8077
8.45	7.04	−0.475	−0.985	0.51	1.37	2.06	0.6656
8.33	8.33	−0.407	−1.035	0.628	3.52	1.61	2.1880
**[M]** **X10** ^**− 4**^ **Mol.L** ^**− 1**^	**[L]** **X10** ^**− 4**^ **Mol.L** ^**− 1**^	**E˚** **volt**	**Da** **X10** ^**− 10**^ **Cm**^**2**^.**s**^**− 1**^	**Dc** **X10** ^**− 9**^ **Cm**^**2**^.**s**^**− 1**^	**Epc/2**	**αnac**	**ks**
8.96	1.49	−0.755	2.00805	10.09	−1.008	1.8651	0.1269
8.82	2.94	−0.759	4.24786	8.57	−0.982	0.7577	0.0837
8.70	4.35	−0.759	4.71559	3.61	−0.982	0.7950	0.0525
8.57	5.71	−0.7655	5.6047	4.39	−0.951	0.4408	0.0538
8.45	7.04	−0.73	7.18684	8.29	−0.939	1.0542	0.0526
8.33	8.33	−0.721	40.87845	5.21	−0.94	0.5104	0.0898

**Table 6:** Cyclic voltammetric data of Quercetin with ferrous ions in supporting electrolyte of KCl (0.1 M) at varies scan rates.

Scan rate	Ep, a volt	Ep, c volt	∆Ep volt	(-)Ip, a X10^4^ Amp	Ip, c X10^4^ Amp	Ip, a/Ip, c
0.10	−0.403	−1.037	0.634	3.38	1.60	2.1142
0.05	−0.52	−1.181	0.661	5.52	4.01	1.3758
0.02	−0.545	−1.15	0.605	7.19	5.60	1.2830
0.01	−0.51	−1.193	0.683	9.37	6.55	1.4314
**Scan rate**	**E˚** **volt**	**Da** **X10** ^**7**^ **Cm**^**2**^.**s**^**− 1**^	**Dc** **X10** ^**7**^ **Cm**^**2**^.**s**^**− 1**^	**Epc/2**	**αnac**	**Ks x 10** ^**− 1**^
0.10	−0.72	0.2175	0.0487	−0.939	0.4948	0.905
0.05	−0.8505	1.1613	0.6135	−1.133	1.0103	4.21
0.02	−0.8475	4.9309	2.9956	−0.914	0.2055	1.55
0.01	−0.8515	16.765	8.1825	−1.11	0.5842	6.45

**Table 7:** Kinetic parameters (D, Ks, α, E°) for ferrous ammonium sulfate and iron(II)-quercetin complex.

system	D (×10⁻⁹ cm²/s)	ks (cm/s)	Α	E° (V)
Fe(II)	8.83	1.865	0.127	−0.755
Fe(II)-quercetin complex	5.21	0.510	0.089	−0.721

### DFT calculations of Quercetin and Fe(II)-Quercetin complex

 Density Functional Theory (DFT) calculations were performed to evaluate and compare the thermodynamic parameters of quercetin and its Fe(II) complex using the Gaussian 09 software. These calculations included total thermal energy, heat capacity, and entropy, along with partition functions and vibrational frequency analysis. The molecular systems studied contain 15 carbon atoms, and the relevant data are summarized in (Tables S6–S8) (Supporting Information).

Thermal Energy Parameters: The computed total thermal energy (E), heat capacity at constant volume (Cv), and entropy (S) for the Fe(II)-quercetin complex (Table 8, and Fig. S6) were found to be 74.104 kcal/mol, 60.243 cal/mol·K, and 120.297 cal/mol·K, respectively. These values indicate substantial contributions from molecular motions. The detailed breakdown of these contributions is as follows:

- Electronic contributions: The electronic energy and associated heat capacity are negligible at standard temperatures.
- Translational and rotational contributions: Both contribute equally (0.889 kcal/mol and 2.981 cal/mol·K each), as expected for small organic molecules.
- Vibrational contributions (Tables S9, S10): These represent the majority of the thermal energy, with values of 72.327 kcal/mol and 54.281 cal/mol·K.

Partition Function Analysis: The partition functions (Q) provide insight into the statistical thermodynamic behavior of the system:

- The total partition function at the ground state (QBot) was 0.344552 × 10⁻²⁸, whereas the value at V = 0 was 0.110772 × 10²⁰, indicating the accessibility of higher energy states.
- The vibrational partition function (Qvib Bot) was extremely low (0.873621 × 10⁻⁴⁴), consistent with low occupancy of vibrational states at ambient temperature.

Vibrational Frequency Distribution: The vibrational frequencies for the quercetin complex revealed mode-dependent contributions to thermal energy. Lower modes displayed higher energy values and higher statistical weights, while higher modes contributed less, as reflected by decreasing partition function values (Q, Log10(Q), Ln(Q)). These trends provide critical insight into how vibrational motion influences molecular stability.

**Table 8:** Thermodynamic comparison between Quercetin and its Fe(II) Complex.

Parameter	Quercetin	Quercetin complex
E (Thermal) [KCal/Mol]	64.313	74.104
CV [Cal/Mol-Kelvin]	68.330	60.243
S [Cal/Mol-Kelvin]	129.538	120.297
Electronic	0.000	0.000
Translational	0.889	0.889
Rotational	0.889	0.889
Vibrational	62.536	72.327

The Fe(II)-quercetin complex shows a higher thermal energy and vibrational contribution than quercetin alone, suggesting more significant intramolecular interactions and possibly enhanced thermal stability. Conversely, quercetin exhibits a higher entropy and heat capacity, implying greater conformational freedom and molecular flexibility.

Frontier Molecular Orbital (FMO) Analysis Quantum chemical descriptors such as HOMO (Highest Occupied Molecular Orbital), LUMO (Lowest Unoccupied Molecular Orbital), ionization potential, electron affinity, and electrophilicity index were computed using the B3LYP/6-311G(d, p) level of theory. The HOMO–LUMO energy gap of the Fe(II)-quercetin complex (0.013 eV) was smaller than that of quercetin (0.019 eV) (Table 9), indicating enhanced chemical reactivity and potential for electron transfer processes^56^. The resultant 3D plots, presented in (Fig. S7), depict distinct orbitals of molecules.

**Table 9:** CalculatedquantumchemicalparametersofquercetinandFe (II)-quercetin complex atB3LYP/6–31G(d, p)levelof theory.

ChemicalParameters	Quercetin(eV)	Fe (II)-quercetin complex(eV)
EHOMO	−0.282	−0.267
ELUMO	−0.263	−0.254
EnergyGap	0.019	0.013
IonisationPotential(IP)	0.282	0.267
ElectronAffinity(EA)	0.263	0.254
ChemicalPotential(µ)	−0.2725	−0.2605
Electronegativity(χ)	0.2725	0.2605
Chemicalhardness(η)	0.0095	0.0065
Chemicalsoftness	105.26	153.85
ElectrophilicityIndex	3.91	5.22

From this data, it can be concluded that the Fe(II)-quercetin complex exhibits a narrower band gap, implying greater chemical reactivity and improved electrical conductivity. The lower ionization potential (IP) and electron affinity (EA) suggest easier electron donation and acceptance, which can enhance redox and antioxidant behaviors. The less negative chemical potential reflects lower resistance to changes in electron population. Moreover, the slight reduction in electronegativity implies the complex is a more efficient electron donor. The lower chemical hardness (η) and increased softness (S) indicate greater chemical adaptability, while the elevated electrophilicity index (ω) highlights the complex’s potential for electron-accepting processes, making it promising for biological and catalytic applications. In conclusion, the DFT results underscore significant differences in electronic and thermodynamic properties between quercetin and its Fe(II) complex. These differences support the enhanced functionality of the complex, particularly in antioxidant and pharmaceutical contexts.

## Conclusion

In this study, the electrochemical behavior of quercetin and ferrous ammonium sulfate in potassium chloride supporting electrolyte at 303.05 K was systematically investigated. Cyclic voltammetry using a glassy carbon electrode revealed that quercetin undergoes irreversible oxidation, while Fe(II) ions exhibit quasi-reversible redox characteristics. Upon complexation in a 1:1 stoichiometric ratio, an Fe(II)-quercetin complex was successfully formed, as evidenced by continuous variation analysis, bathochromic shifts in UV-Vis spectra, and pronounced irreversibility in the voltammetric response. A charge transfer coefficient (α) below 0.3 further supports the formation of a stable coordination complex. The synthesized Fe(II)-quercetin complex exhibited enhanced biological activities compared to the free components. In vitro cytotoxicity assays demonstrated significantly increased antiproliferative effects against HepG2 and MCF-7 cancer cell lines. The antioxidant activity of quercetin was significantly enhanced upon complexation with Fe(II), as evidenced by the lower IC₅₀ value of the complex (21.86 µg/mL) compared to free quercetin, indicating improved radical scavenging efficiency due to metal-induced stabilization and enhanced electron-donating capacity. These findings were further corroborated by molecular docking studies targeting the SARS-CoV-2 spike protein receptor (PDB ID: 7JWY), where the Fe(II)-quercetin complex showed a stronger binding affinity (i.e., lower total binding energy) than quercetin alone, suggesting favorable interactions and potential antiviral properties. Density Functional Theory (DFT) calculations revealed notable differences in the physicochemical properties of the free ligand and its metal complex. The Fe(II)-quercetin complex exhibited a smaller HOMO–LUMO energy gap, greater softness, and higher electrophilicity index, indicating increased chemical reactivity and electron-accepting capacity. Moreover, the complex demonstrated higher thermal energy and vibrational contributions, whereas free quercetin showed higher heat capacity and entropy, reflecting distinct thermodynamic behaviors and molecular stabilities. Collectively, these results highlight the multifunctional potential of the Fe(II)-quercetin complex as a promising candidate for antioxidant therapy, cancer treatment, and antiviral drug development. This study also underscores the critical role of flavonoid-metal interactions in modulating electrochemical properties and enhancing biological performance, offering a viable strategy for designing bioactive metal–flavonoid complexes with pharmaceutical and biomedical relevance.

## Supplementary Information

Below is the link to the electronic supplementary material.

Supplementary Material 1

## Data Availability

All data generated or analysed during this study are included in this published article [and its supplementary information files].
