# Supplementary material for: Redox properties of quercetin iron II complex with enhanced antioxidant and antiviral activities
Source: Sci Rep. 2025 Dec 19;15:44139. doi: 10.1038/s41598-025-31433-z (PMC12717270; doi:10.1038/s41598-025-31433-z)
Supplement: Supplementary file 1 — Supplementary Material 1 [file 41598_2025_31433_MOESM1_ESM.docx]

Redox Properties of Quercetin Iron II Complex with Enhanced Antioxidant and Antiviral Activities

Sara E Abdel Hameed^1^, Weam M. Abou El-Maaty^1^, Esam A. Gomaa^1^, Fathi S. Awad^1, *^

Chemistry Department Faculty of Science, Mansoura University, Mansoura, Egypt

**Supporting Information**


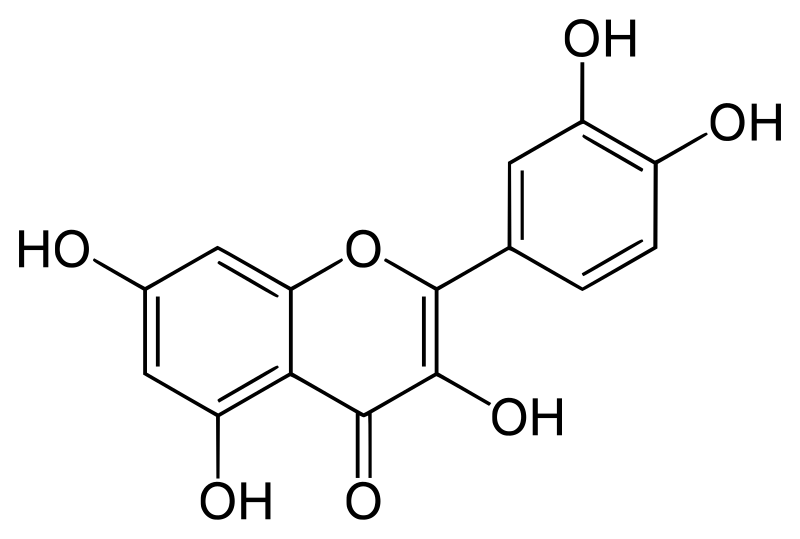


**Fig.S1.**Quercetin structure

**Table S1. Average of Relative viability of cells (%)**

| Samples | 100(µM) | 50(µM) | 25(µM) | 12.5(µM) | 6.25(µM) | 3.125(µM) | 1.56(µM) |
| --- | --- | --- | --- | --- | --- | --- | --- |
| DOX(MCF-7) | 6.2 | 10.9 | 14.3 | 26.9 | 41.5 | 58.4 | 69.1 |
| DOX(HePG-2) | 6.3 | 11.2 | 14.1 | 28.3 | 45.8 | 57.6 | 71.2 |
| SOR(MCF-7) | 8.1 | 15.2 | 23.0 | 34.8 | 51.6 | 68.9 | 87.5 |
| SOR(HePG-2) | 9.2 | 16.6 | 25.7 | 37.2 | 61.3 | 72.9 | 95.4 |
| Ferrous ammonium sulfate(MCF-7) | 41.1 | 49.8 | 66.4 | 79.1 | 92.7 | 100 | 100 |
| Ferrous ammonium sulfate(HePG-2) | 43.3 | 57.2 | 69.7 | 85.4 | 99.2 | 100 | 100 |
| quercetin(MCF-7) | 8.9 | 17.9 | 26.8 | 33.4 | 62.6 | 71.2 | 89.9 |
| quercetin(HePG-2) | 7.8 | 13.2 | 20.0 | 33.2 | 49.3 | 64.3 | 87.3 |
| Fe(II)-quercetin complex(MCF-7) | 8.0 | 20.3 | 22.3 | 39.4 | 57.9 | 84.6 | 97.3 |
| Fe(II)-quercetin complex (HePG-2) | 9.2 | 17.2 | 25.4 | 31.3 | 61.5 | 69.2 | 89.1 |

**Table S2.**Cytotoxicity (IC_50_)of tested compounds on two different cell lines.

| **No.** | **Compound** | **In vitro Cytotoxicity IC50 (µM)*** | |
| --- | --- | --- | --- |
|  |  | **MCF-7** | **HePG-2** |
| ****** | **DOX** | **4.17±0.2** | **4.50±0.2** |
| ******* | **SOR** | **7.26±0.3** | **9.18±0.6** |
| **1** | **Ferrous ammonium sulfate** | **81.34±4.1** | **91.81±4.7** |
| **2** | **Fe (II)-Quercetin complex** | **18.60±1.4** | **26.80±1.7** |
| **3** | **Quercetin** | **9.75±0.8** | **8.14±0.5** |

***IC50 (µM)**: 1 – 10 (very strong). 11 – 20 (strong). 21 – 50 (moderate). 51 – 100 (weak) and above 100 (non-cytotoxic)

**** DOX**: Doxorubicin

*****SOR**: Sorafenib


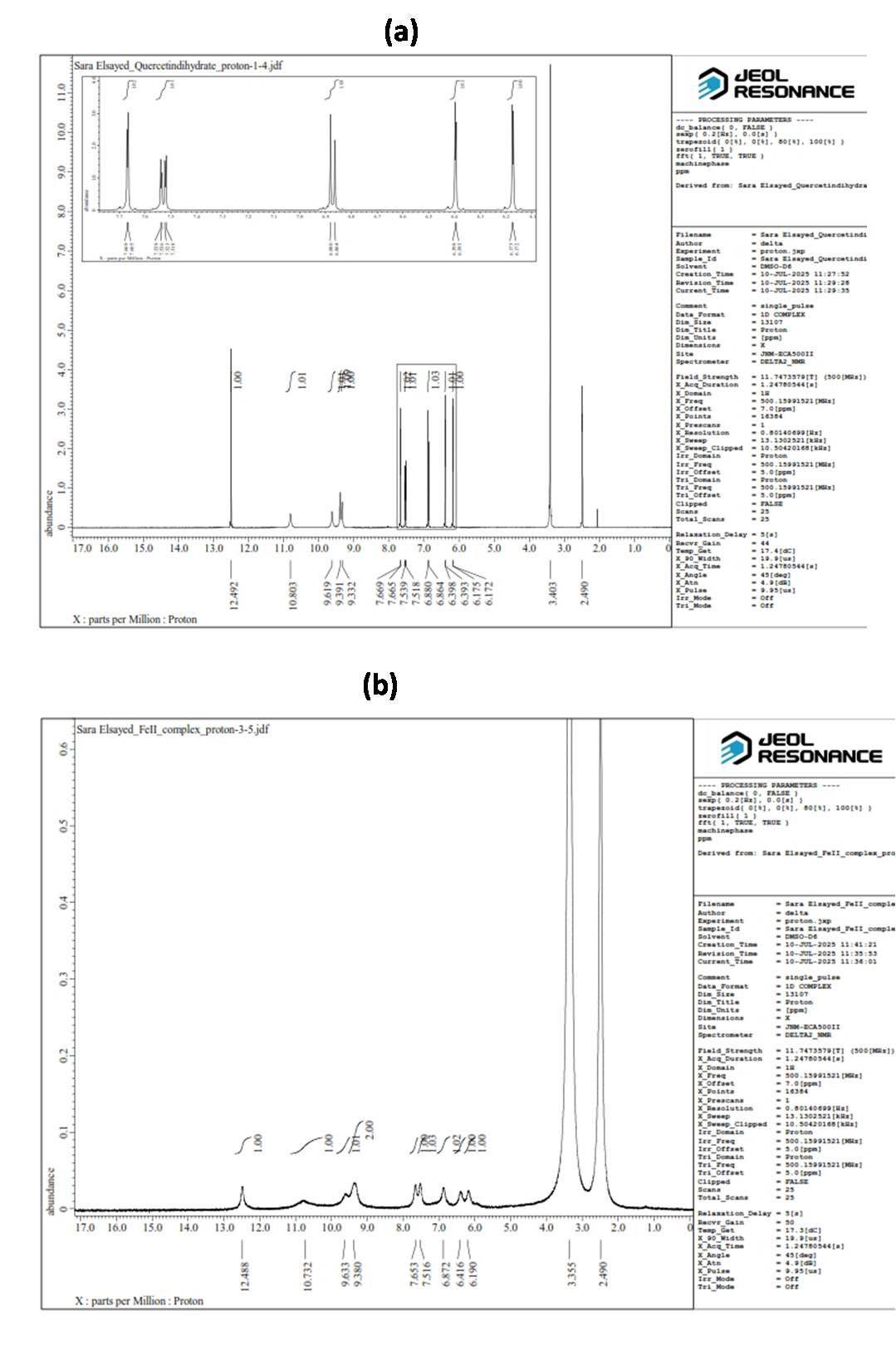


**Fig.S2.** ¹H NMR spectra for (a) quercetin and (b) Fe (II)-quercetin complex

**Fig.S3.** Cyclic voltammetry of (0.01M) quercetin with scan rate 0.1V/s

**Table S3.**The docking interaction parameters of quercetin with 7JWY: VIRAL PROTEIN / 7JWYCOVID -19.

| **Ligand** | **Receptor** | | **Interaction** | **Distance** | **E (kcal/mol)** | **S** | **rmsd** |
| --- | --- | --- | --- | --- | --- | --- | --- |
| O22 31 | O SER 371 (A) | | H-donor | 2.72 | -3.9 | -5.4246 | 2.9533 |
| O20 28 | CA GLY 339 (A) | | H-acceptor | 3.41 | -0.8 |  |  |
| **Quercetin Interactions ACE2 receptor Report (VIRAL PROTEIN/7JWY)** | | | | | |  |  |
| **Ligand** | | **Receptor** | **Interaction** | **Distance** | **E (kcal/mol)** |  |  |
| O29 | | THR1077 (A) | H-donor | 2.84 | -3.6 | -4.7315 | 1.8013 |

**
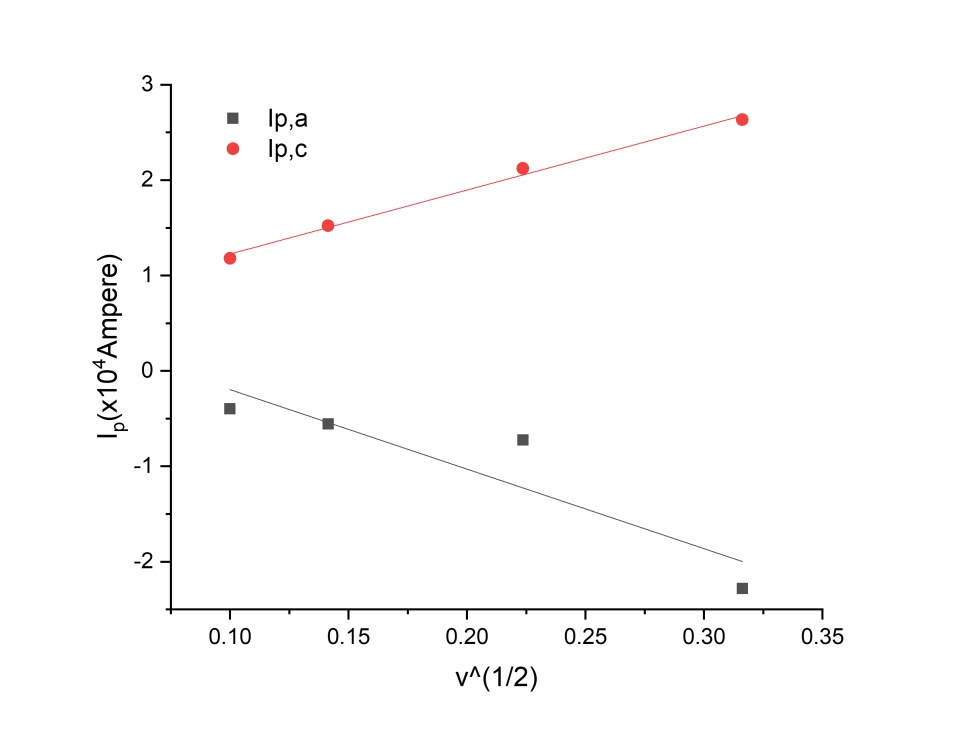
**

**Fig.S4.** The relation between peak current and square root of the scan rates for ferrous ammonium sulfate at 303.05K

**Table S4.** Kinetic and solvation parameters of iron (II)

| Scan rate | Γc  X10^7^  Mol.s^-2^ | (+) Qc  X10^4^ C | Γ a  X10^8^  Mol.s^-2^ | (-) Q a  x10^4^ |
| --- | --- | --- | --- | --- |
| **0.10** | 0.908 | 2.75 | 7.867 | 2.38 |
| **0.05** | 1.463 | 4.43 | 4.998 | 1.51 |
| **0.02** | 2.626 | 7.96 | 2.698 | 2.91 |
| **0.01** | 4.069 | 12.30 | 13.738 | 4.16 |

**
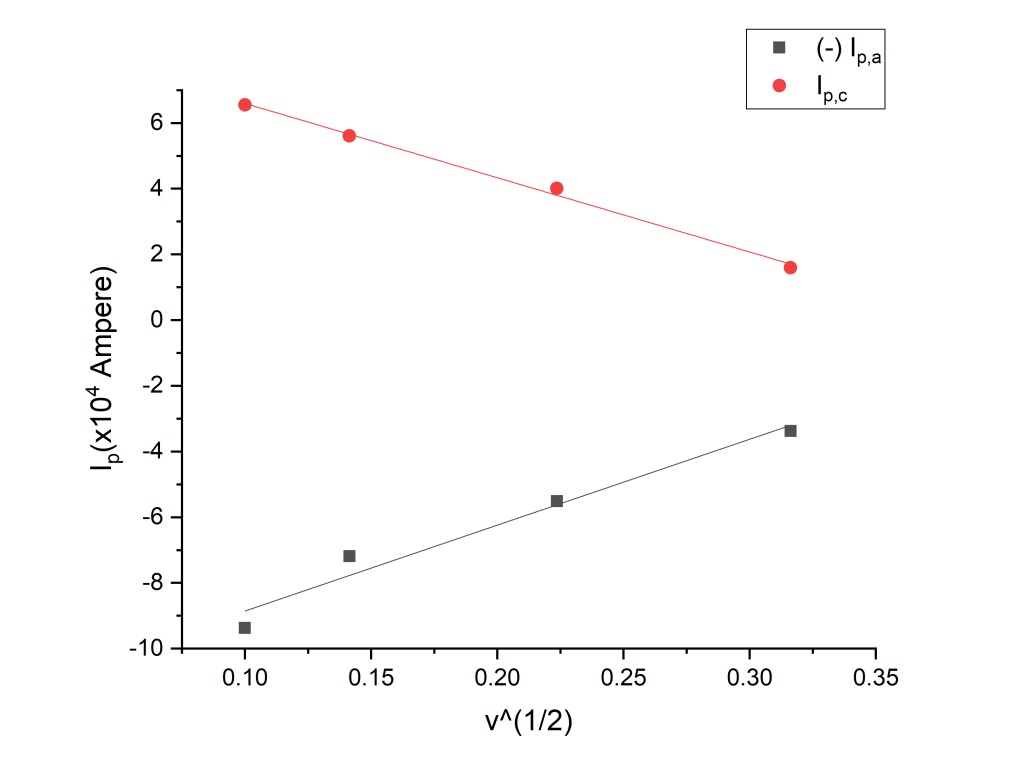
**

**Fig.S5.** The relation between peak current and square root of the scan rates for quercetin on ferrous ions at 303.05K

**Table S5.**Cyclic voltammetry data (Gibbs free energy and stability constants) of quercetincomplexation interaction with ferrous ions

| M x 10^-4^  Ammonium  ferrous sulfate | M x 10^-4^  Quercetin | Log β_MX_ | β_MX_ | ∆G KJ/mol |
| --- | --- | --- | --- | --- |
| 8.96 | **1.49** | **1.4109** | **25.7565** | **-8.1867** |
| 8.82 | **2.94** | **2.0169** | **103.9632** | **-11.7030** |
| 8.70 | **4.35** | **2.5206** | **331.5765** | **-14.6258** |
| 8.57 | **5.71** | **3.1010** | **1287.7393** | **-18.0449** |
| 8.45 | **7.04** | **2.9844** | **964.7426** | **-17.3172** |
| 8.33 | **8.33** | **3.2870** | **1936.5677** | **-19.0731** |

**Table S6. Theoretical thermal energy parameters for quercetin**

**E (Thermal) CV S**

KCal/MolCal/Mol-Kelvin Cal/Mol-Kelvin

Total 64.313 68.330 129.538

Electronic 0.000 0.000 0.000

Translational 0.889 2.981 42.912

Rotational 0.889 2.981 34.910

Vibrational 62.536 62.368 51.716

Vibration 1 0.597 1.971 4.288

Vibration 2 0.599 1.965 3.994

Vibration 3 0.607 1.940 3.236

Vibration 4 0.613 1.918 2.865

Vibration 5 0.621 1.893 2.566

Vibration 6 0.629 1.866 2.323

Vibration 7 0.634 1.851 2.210

Vibration 8 0.637 1.841 2.144

Vibration 9 0.654 1.789 1.849

Vibration 10 0.666 1.752 1.685

Vibration 11 0.673 1.733 1.612

Vibration 12 0.692 1.675 1.422

Vibration 13 0.694 1.669 1.403

Vibration 14 0.702 1.647 1.341

Vibration 15 0.717 1.604 1.232

**Table S7. Theoretical thermal energy parameters for Fe (II)-quercetin complex**

**E (Thermal) CV S**

KCal/Mol Cal/Mol-Kelvin Cal/Mol-Kelvin

Total 74.104 60.243 120.297

Electronic 0.000 0.000 0.000

Translational 0.889 2.981 43.633

Rotational 0.889 2.981 35.678

Vibrational 72.327 54.281 40.985

**Table S8. Partition Functions Q and Vibrational Frequencies for Fe(II)-quercetin complex**

**Q Log10(Q) Ln(Q)**

Total Bot 0.344552D-28 -28.462745 -65.537893

Total V=0 0.110772D+20 19.044429 43.851419

Vib (Bot) 0.873621D-44 -44.058677 -101.448852

Vibration 1 0.608 1.936 3.154

Vibration 2 0.612 1.922 2.920

Vibration 3 0.619 1.899 2.636

Vibration 4 0.630 1.866 2.319

Vibration 5 0.638 1.839 2.127

Vibration 6 0.652 1.795 1.878

Vibration 7 0.662 1.764 1.737

Vibration 8 0.663 1.761 1.722

Vibration 9 0.671 1.737 1.627

Vibration 10 0.680 1.711 1.536

Vibration 11 0.688 1.686 1.454

Vibration 12 0.698 1.659 1.374

Vibration 13 0.703 1.642 1.328

Vibration 14 0.730 1.566 1.146

Vibration 15 0.735 1.554 1.121


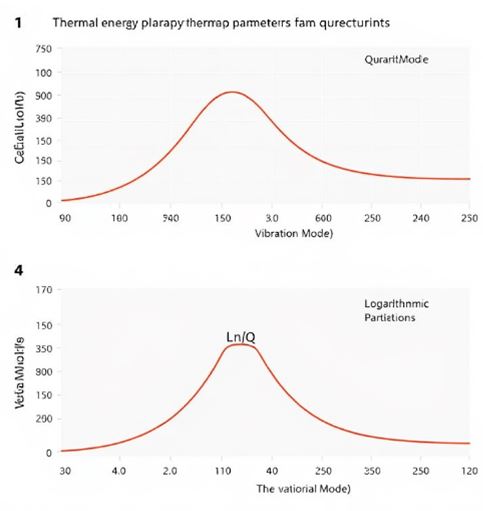


**Fig.S6. The thermal energy parameters and the logarithmic partition functions curve for quercetin complex based on DFT calculations.**

**Table S9. Quercetin Vibrational Modes**

| **Vibration** | **Frequency [KCal/Mol]** | **CV [Cal/Mol-Kelvin]** | **S [Cal/Mol-Kelvin]** |
| --- | --- | --- | --- |
| 1 | 0.597 | 1.971 | 4.288 |
| 2 | 0.599 | 1.965 | 3.994 |
| 3 | 0.607 | 1.940 | 3.236 |
| ... | ... | ... | ... |
| 15 | 0.717 | 1.604 | 1.232 |

**Table S10. Quercetin Complex Vibrational Modes**

| **Vibration** | **Frequency [KCal/Mol]** | **CV [Cal/Mol-Kelvin]** | **S [Cal/Mol-Kelvin]** |
| --- | --- | --- | --- |
| 1 | 0.608 | 1.936 | 3.154 |
| 2 | 0.612 | 1.922 | 2.920 |
| 3 | 0.619 | 1.899 | 2.636 |
| ... | ... | ... | ... |
| 15 | 0.735 | 1.554 | 1.121 |

| 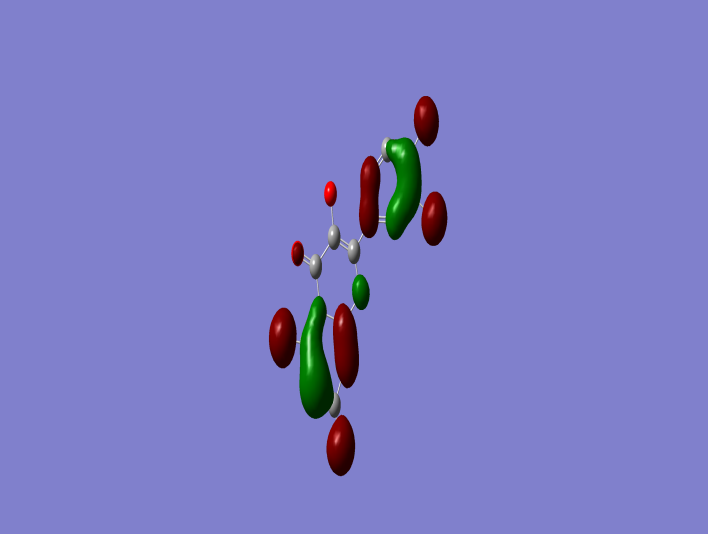 | 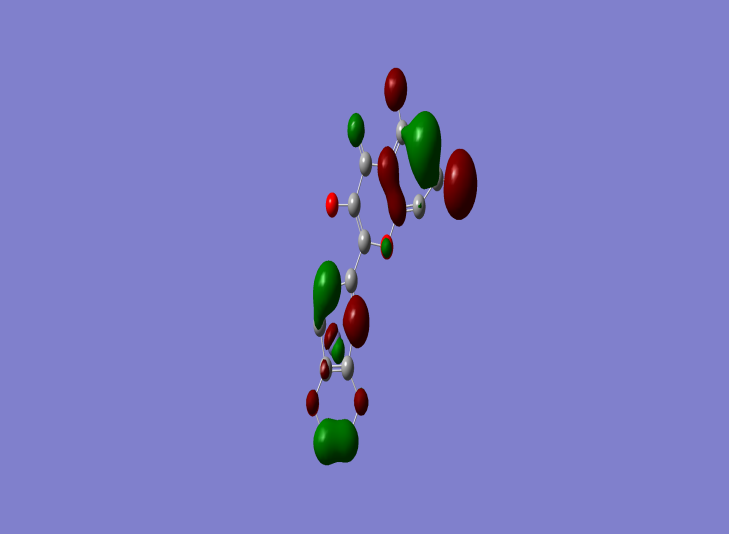 |
| --- | --- |

**Fig.S7.** Frontier Molecular Orbitals of the quercetin and Fe (II)-quercetin complex, computed at B3LYP/6-311G(d,p).
